# The Thirty-Day Mortality Rate and Nephrotoxicity Associated With Trough Serum Vancomycin Concentrations During Treatment of Enterococcal Infections: A Propensity Score Matching Analysis

**DOI:** 10.3389/fphar.2021.773994

**Published:** 2022-01-28

**Authors:** Wasan Katip, Siriporn Okonogi, Peninnah Oberdorfer

**Affiliations:** ^1^ Department of Pharmaceutical Care, Faculty of Pharmacy, Chiang Mai University, Chiang Mai, Thailand; ^2^ Research Center for Pharmaceutical Nanotechnology, Faculty of Pharmacy, Chiang Mai University, Chiang Mai, Thailand; ^3^ Department of Pharmaceutical Sciences, Faculty of Pharmacy, Chiang Mai University, Chiang Mai, Thailand; ^4^ Division of Infectious Diseases, Department of Pediatrics, Faculty of Medicine, Chiang Mai University, Chiang Mai, Thailand

**Keywords:** therapeutic drug monitoring, vancomycin, enterococcal infections, vancomycin trough levels, mortality rate

## Abstract

The objective of this study was to evaluate the relationship between vancomycin trough levels in patients with documented enterococcal infections and mortality, clinical outcomes, microbiological outcomes, and nephrotoxicity. We conducted a retrospective cohort study of patients with *enterococcus* infections who were prescribed vancomycin with therapeutic drug monitoring during January 2010 and December 2019 at Chiang Mai University Hospital (CMUH). The study enrolled 300 participants who met the inclusion criteria and were prescribed vancomycin with therapeutic drug monitoring. The results of this study showed that, after propensity score matching, a vancomycin trough of ≥15 mg/L was associated with significant differences in 30-days mortality compared to a vancomycin trough of <15 mg/L (aHR: 0.41, 95% CI: 0.21–0.82; *p* = 0.011). Likewise, a vancomycin trough of ≥15 mg/L was associated with significant differences in the clinical response (aHR: 0.49, 95% CI: 0.26–0.94; *p* = 0.032), microbiological response (aHR: 0.32, 95% CI: 0.12–0.87; *p* = 0.025) and nephrotoxicity (aHR: 3.17, 95% CI: 1.39–7.23; *p* = 0.006), compared with a vancomycin trough of <15 mg/L. However, sub-group analysis found that very high trough levels (>20 mg/L) were also associated with a high rate of nephrotoxicity (aHR: 3.55, 95% CI 1.57–8.07, *p* = 0.002), when compared with a vancomycin trough of <15 mg/L. The target vancomycin trough concentration was ≥15 mg/L and this target can be an optimal alternative to the use of area under the curve (AUC) values for monitoring the treatment of enterococcal infection.

## Introduction

*Enterococci* spp., especially *Enterococcus faecium* and *Enterococcus feacalis* are Gram positive, facultative anaerobe, oval, cocci bacteria, which form chains of varying lengths (Schröder et al., 2015). They are present naturally in gastrointestinal system colonisers, but can also cause a number of infections, including pelvic infections, endocarditis, wound infections, and urinary tract infections (UTIs). These range from uncomplicated infections to sepsis, which may be life-threatening and is of major concern worldwide (Schröder et al., 2015). In Thailand, the prevalence of penicillin-resistant *Enterococcus* spp*.* has increased, from 42.1% in 2010 to 50.5% in 2020 (NARST, 2021). Among the therapeutic options, vancomycin is a glycopeptide antibiotic widely used for treatment of Methicillin-resistant *Staphylococcus aureus* (MRSA), Methicillin-Resistant *Staphylococcus epidermidis* (MRSE) and *E. faecalis* and *E. faecium* infections*.* The response to vancomycin is concentration-independent (Moise-Broder et al., 2004; Deryke and Alexander, 2009). Moreover, the pharmacokinetic (PK)/pharmacodynamic (PD) parameter that correlate best with favorable outcomes of invasive MRSA infections are based on the area under the curve of the vancomycin concentration-time graph above the minimum inhibitory concentration (AUC/MIC) (Kullar et al., 2011a; Matsumoto et al., 2013). However, it may be difficult to obtain multiple serum concentrations to determine the AUC in practice. Thus, several guidelines recommend trough concentration monitoring as a practical surrogate marker (Deryke and Alexander, 2009; Matsumoto et al., 2013). Furthermore, the clinical measurement of AUC included analysis of several serum vancomycin concentrations at the same dosing interval, requiring use of PK software which was not readily accessible at all institutions. As such, in clinical practice found that trough-directed dosing is a more realistic alternative to AUC/MIC-guided dosing (Deryke and Alexander, 2009; Matsumoto et al., 2013).

There are several knowledge gaps surrounding the clinical use and effectiveness of vancomycin in patients with enterococcal infection, but it continues to be used in these patients due to a lack of data (Rybak et al., 2009; Matsumoto et al., 2013; Rybak et al., 2020). Moreover, there is no clear correlation between trough concentrations of vancomycin and the eradication of enterococcal infection or good patient outcomes (Jumah et al., 2018; Nakakura et al., 2019). In addition, the risk of adverse outcomes, such as nephrotoxicity, may be increased by increasing vancomycin serum concentrations. While the possible increased risk of nephrotoxicity is mentioned in a recently published consensus paper, the authors emphasise that there is minimal data availability (Gawronski et al., 2013; van Hal et al., 2013). The aim of this study was to determine whether mortality, clinical and microbiological outcomes, and vancomycin-related nephrotoxicity in patients with *Enterococcus* infections are correlated with vancomycin trough concentrations at the initial steady state. Moreover, we aim to compare primary and secondary outcomes for enterococcal infection patients with vancomycin trough concentration ≥15 mg/L and AUC/MIC≥ 389 mg*hr/L.

## Materials and Methods

This retrospective study was conducted in all patients treated with intravenous vancomycin as in patients at Chiang Mai University Hospital (CMUH, a university-affiliated hospital located in Chiang Mai, Thailand) between January 2010 and December 2019. This study was approved by the ethics committee on human research of the Faculty of Medicine, Chiang Mai University (NONE-2563-07835), with a waiver of informed consent for retrospective data collection, under the condition that data is stored anonymously. The criteria used to identify and classify infections were taken from the Center for Disease Control and Prevention (CDC) (Horan et al., 2008), according to the evaluations of infectious disease (ID) physicians.

The inclusion criteria were: 1) age over 18 years; 2) treatment with vancomycin for more than 3 days in patients with microbiological confirmation of infection with *Enterococci* spp. susceptible to vancomycin (for patients with more than one episode of enterococcal infection, only the first episode during the study period was included); and 3) at least one measurement of serum concentrations of vancomycin and appropriate measurement of trough level. Patients were excluded from the study if they were: 1) exposed to intravenous vancomycin within the last 7 days, 2) pregnant, or 3) undergoing haemodialysis, 4) had polymicrobial infection, or infection with isolates resistant to vancomycin, 5) patients with *Enterococcus* cultures assessed to be colonisers or contaminants, 6) who had incomplete patient records, were also excluded.

Vancomycin was given as a 60-min i.v. infusion [in patients with normal renal function, vancomycin at a loading dose of 30 mg/kg (1.5 g) the maintenance dose of 20 mg/kg (1 g) every 12 h]. In the case of renal dysfunction, the doses were modified, following the suggestions of the CMUH guide protocol. The CMUH protocol was initiated by the pharmacy and infectious disease physician (The Pharmacy and Therapeutics Committee), written in the CMU antibiotic guidebook and is recommended for use in the hospital (Micromedex, 2015; Corbett et al., 2016; CMU antibiotic guidebook, 2018). In patients with renal dysfunction, the vancomycin dosage was adjusted based on their kidney function (creatinine clearance; CL_CR_ ≥ 50 ml/min: 1 g every 12 h, CL_CR_ 40–49: 750 mg every 12 h, CL_CR_ < 40 ml/min: 750 g every 48–120 h) (CMU antibiotic guidebook, 2018).

### Data Collection

Data from patient medical records and computerised hospital databases were collected. The data collected included age, gender, body mass index, duration of vancomycin treatment, underlying disease (cerebrovascular disease, solid tumor, hematologic malignancy, chronic kidney disease (CKD) is defined as a glomerular filtration rate (GFR) of less than 60 ml/min/1.73 m^2^ for more than 3 months, regardless of cause (Levey et al., 2005), chronic liver disease, coronary artery disease, or diabetes mellitus), length of hospital stay, APACHE II score, baseline serum creatinine, types of nephrotoxic medications, source of *Enterococcus* infection, enterococcal species, mortality status and bacteriological data. The trough levels were defined as the vancomycin concentration measured shortly before the next dose. Initial vancomycin trough concentration was measured at steady-state (before the fourth dose of vancomycin administration), and not more than 1 week after commencing vancomycin therapy, as suggested by the guidelines (Deryke and Alexander, 2009; Matsumoto et al., 2013). To measure the trough level appropriately, serum had to be collected within 30 min of the next dose (Roustit et al., 2010).

### The AUC_0–24_/MIC Ratio Calculation

PrecisePK software (Healthware Inc., San Diego, CA, United States) was used to calculate the AUC on day 1 (AUC_0–24_) after treatment using the Bayesian theorem. The Bayesian conditional posterior of patient pharmacokinetic parameters adjusted by individual vancomycin serum concentrations was estimated using the one-compartment model and population parameters (Bayesian prior values) included in PrecisePK (s). The AUC_0-24_/MIC was calculated using the automated microbiology system VITEK 2 MIC technique.

### Statistical Analyses

For statistical analysis, the study sample was divided into two groups: patients with a vancomycin trough level of <15 mg/L (the control group) and patients with a vancomycin trough level of ≥15 mg/L (the intervention group). Moreover, patients were also divided into two groups with a vancomycin AUC/MIC of < 389 mg*hr/L (the control group) and patients with a vancomycin AUC/MIC of ≥ 389 mg*hr/L (the experimental group). Continuous variables are expressed as means and standard deviations and were compared using the Student’s t-test. Categorical variables were expressed as frequencies and percentages, and were compared using Fisher’s exact test where appropriate. We used the propensity score (PS) method to minimise the potentially substantial bias between the two groups, since the patients in our study were not randomised.

In order to determine univariate variables associated with the vancomycin trough level, patients who met the inclusion criteria were analysed. Using univariate variables with a *p*-value of 0.20 or less, a nominal logistic multivariate analysis was used to evaluate independent variables associated with the vancomycin trough level. The variables included in the PS calculation were age, gender, APACHE II score, serum albumin, *Enterococcus* species, colistin, duration of vancomycin therapy, solid tumor presence, chronic kidney disease, and baseline serum creatinine. A formula was created from this multivariate analysis to determine a propensity score for each individual. The vancomycin trough level <15 mg/L group was then matched by PS matching with the vancomycin trough level ≥15 mg/L group, using a next-nearest approach with a 1:2 ratio (Garrido et al., 2014).

Thirty-day mortality was used as the primary endpoint, with clinical and microbiological outcomes and nephrotoxicity as secondary outcomes. Univariate variables that were associated with 30-days mortality (*p* < 0.20) were entered into a nominal logistic multivariate analysis (along with the vancomycin trough level group and vancomycin AUC/MIC group) to validate the primary endpoint results of the PS-matched groups and describe independent variables associated with the primary endpoint. In addition, time to mortality, clinical and microbiological outcomes, and nephrotoxicity were described using a Cox proportional hazards model.

The risk factors for 30-days mortality among all patients with enterococcal infections were determined using Cox regression analysis. Multivariate Cox proportional hazards model was used to evaluate all variables with *p* < 0.25 in univariate analysis. In addition, at the discretion of the investigators, all variables that showed a trend toward correlation with outcomes were incorporated into the model. Analysis and data interpretation was conducted using Stata software, version 14 (Stata-Corp, College Station, TX). Two-tailed *p-*values < 0.05 were considered statistically significant.

### Outcome Measurement

The primary outcome was the 30-days mortality, defined as death within 30 days of initiation of treatment with vancomycin. The time-to-event for death or discharge was censored. One secondary outcomes of concern were the clinical response to therapy. Clinical response of treatment was assessed by resolution or partial resolution of fever, leukocytosis, and local signs and symptoms of enterococcal infections during treatment with vancomycin. The criteria used to identify and classify infections were taken from the Center for Disease Control and Prevention (CDC) (Horan et al., 2008). Clinical failure was defined as failure resolution of fever, leukocytosis, local signs, and symptoms of enterococcal infections during treatment with vancomycin on days 14. At the end of therapy, microbiological response was characterised by a follow-up of two consecutive *Enterococcus*-negative cultures of clinical samples collected after the initial positive culture from the infection site, while microbiological failure was defined as *Enterococcus* persistence in subsequent cultures of the specimen. Nephrotoxicity was defined as an increase in serum creatinine (SCr) > 0.5 mg/dl above baseline for at least two consecutive days (Deryke and Alexander, 2009). Moreover, changes in serially recorded serum creatinine and urine output were used as KDIGO criteria to characterize the occurrence and phases of AKI with vancomycin treatment (Kidney Disease Improving Global Outcomes, 2012).

### Antimicrobial Susceptibility Test

All isolates were identified as *Enterococcus* spp. using conventional methods at the division of Clinical Microbiology, Chiang Mai University Hospital. The pathogens were considered sensitive to ampicillin or vancomycin if they met the thresholds defined by the Clinical and Laboratory Standards Institute (MIC of ≤8 μg/ml for ampicillin and ≤4 μg/ml for vancomycin) (CLSI, 2015). The species detection and susceptibility tests were performed in the clinical laboratory using the automated microbiology system VITEK 2 (bioMerieux, Inc., Marcy I ’Etoile, France).

## Results

**FIGURE 1:**
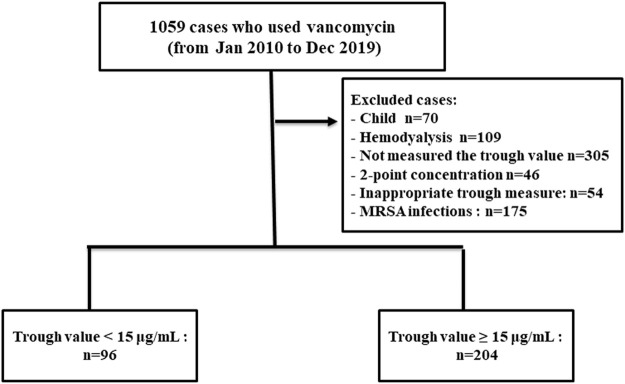
Flowchart of the study population.

**FIGURE 2:**
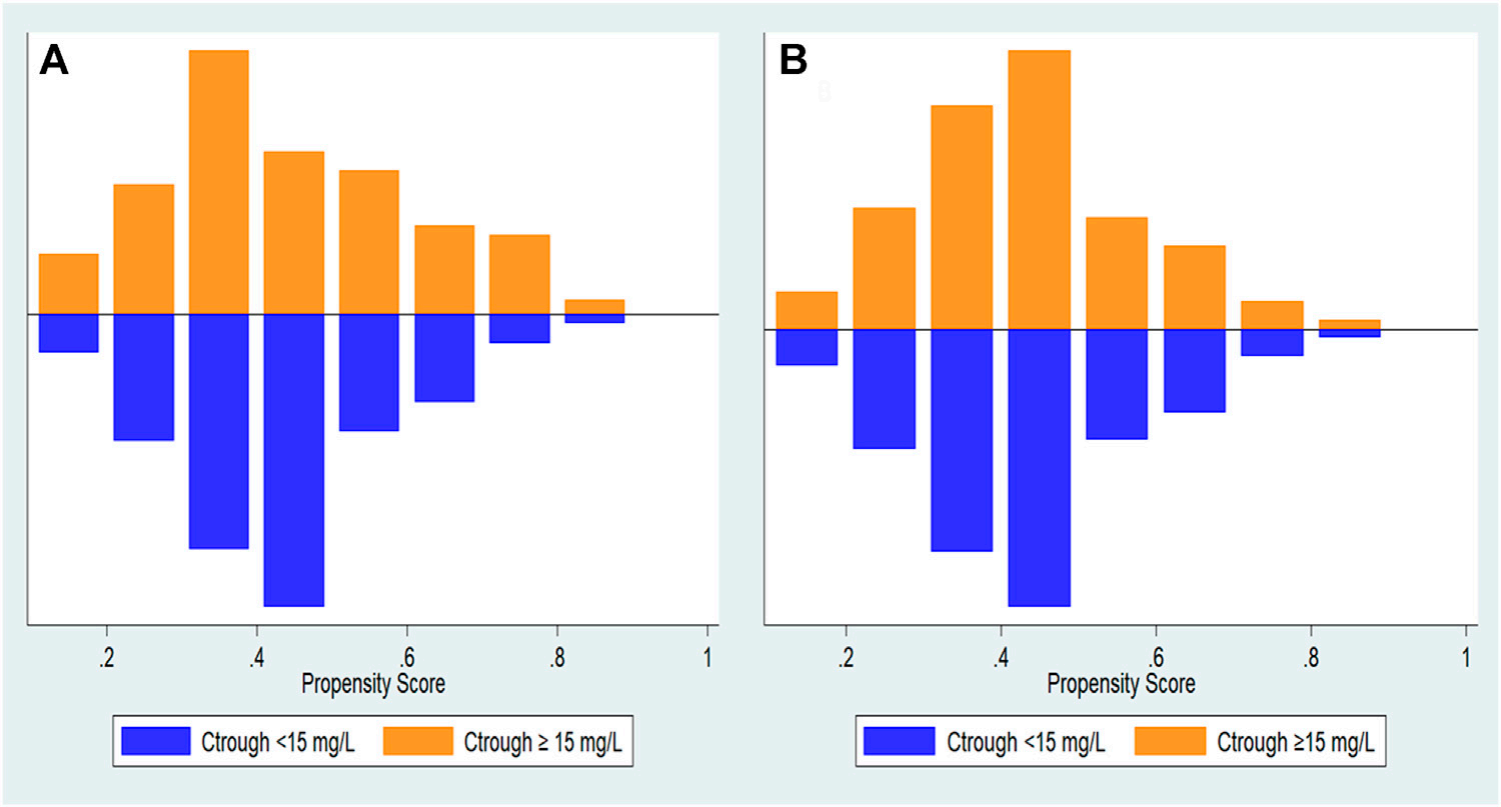
Distribution of propensity scores. **(A)** propensity score before matching and **(B)** propensity score after matching.

During the study period, 300 patients hospitalised with enterococcal infection who fulfilled the inclusion criteria were reviewed. The remaining 300 patients were divided into two groups based on vancomycin trough concentration (Figure 1). The trough levels for most patients took before the fourth dose of vancomycin. One hundred and sixty-eight cases (56.00%) were female and the mean age was 60.89 ± 18.18 years old. Coronary artery disease and chronic kidney disease were the most frequent underlying diseases (Table 1). All 300 *Enterococcus* species from nosocomial infections were resistant to ampicillin but susceptible to vancomycin. *Enterococcus* species strains had a vancomycin MIC value of 4 μg/ml (4 strains), followed by 2 μg/ml (11 strains), 1 μg/ml (235 strains), ≤0.5 μg/ml (50 strains), respectively. Ninety-six patients (32.00%) were in vancomycin trough level group <15 mg/L and 204 patients (68.00%) were in vancomycin trough level group ≥15 mg/L. Comparisons of patient characteristics between the two trough level groups are shown in Table 1. There are differences between two groups on the bacterial species, baseline serum creatinine (Scr) and APACHE II scores. Figure 2 shows the distribution of propensity scores between groups, before and after matching and shows the distribution balance for the variable.

**TABLE 1:** Baseline characteristics of patients with Enterococcal infection treated with vancomycin (n = 300 cases).

Characteristic	Unmatched cohort			Propensity-matched cohort (1:2)			Vancomycin AUC/MIC_vitek_		
	C_trough_ <15 mg/L n = 96	C_trough_ ≥15 mg/L n = 204	*p*-value	C_trough_ <15 mg/L n = 83	C_trough_ ≥15 mg/L n = 166	*p*-value	AUC/MIC <389 mg^a^hr/L n = 60	AUC/MIC ≥389 mg^a^hr/L n = 189>	*p*-value
Male, n (%)	48 (50.00)	84 (41.18)	0.171	41 (49.40)	64 (38.55)	0.105	26 (43.33)	79 (41.80)	0.881
Female, n (%)	48 (50.00)	120 (58.82)		42 (50.60)	102 (61.45)		34 (56.67)	110 (58.20)	
Age, mean ± SD, years	59.40 ± 19.05	61.60 ± 17.76	0.835	60.77 ± 19.17	61.17 ± 18.57	0.875	61.27 ± 18.35	60.96 ± 18.90	0.913
Body mass index (kg/m^2^), mean ± SD	23.54 ± 3.65	23.31 ± 4.55	0.665	23.41 ± 3.43	23.61 ± 4.39	0.694	23.32 ± 4.32	23.21 ± 4.41	0.866
Diagnosis, n (%)									
Catheter-associated urinary tract infection	66 (68.75)	147 (72.06)	0.587	58 (69.88)	123 (74.10)	0.547	47 (78.33)	134 (70.90)	0.319
Bacteremia	10 (10.42)	22 (10.78)	1.000	9 (10.84)	17 (10.24)	1.000	5 (8.33)	21 (11.11)	0.635
Wound infection	14 (14.58)	24 (11.76)	0.577	11 (13.25)	18 (10.84)	0.676	5 (8.33)	24 (12.70)	0.489
Intra-abdominal infection	6 (6.25)	11 (5.39)	0.792	5 (6.02)	8 (4.82)	0.765	3 (5.00)	10 (5.29)	1.000
*Enterococcal* species, n (%)									
*E.faecium*	70 (72.92)	166 (81.37)	0.099	61 (73.49)	141 (84.94)	0.039	45 (75.00)	157 (83.07)	0.186
*E.feacalis*	26 (27.08)	38 (18.63)		22 (26.51)	25 (15.06)		15 (25.00)	32 (16.93)	
Comorbid disease states, n (%)									
Respiratory disease	5 (5.21)	14 (6.86)	0.800	5 (6.02)	14 (8.43)	0.617	3 (5.00)	16 (8.47)	0.577
Cerebrovascular disease	7 (7.29)	15 (7.35)	1.000	6 (7.23)	12 (7.23)	1.000	6 (10.00)	12 (6.35)	0.391
Solid tumor	25 (26.04)	42 (20.79)	0.301	22 (26.51)	34 (20.48)	0.334	17 (28.33)	39 (20.63)	0.219
Hematologic malignancy	1 (1.05)	7 (3.43)	0.443	1 (1.22)	6 (3.61)	0.431	0 (0.00)	7 (3.70)	0.203
Chronic kidney disease	24 (25.00)	76 (37.25)	0.037	22 (26.51)	59 (35.54)	0.196	16 (26.67)	65 (34.39)	0.343
Stage 2	12 (17.39)	45 (22.06)	0.494	12 (14.46)	31 (18.67)	0.479	9 (15.00)	36 (19.05)	0.566
Stage 3	5 (5.21)	12 (5.88)	1.000	5 (6.02)	11 (6.63)	1.000	3 (5.00)	14 (7.41)	0.769
Stage 4	4 (4.17)	10 (4.90)	1.000	3 (3.61)	10 (6.02)	0.553	3 (5.00)	9 (4.76)	1.000
Stage 5	3 (3.13)	9 (4.41)	0.758	2 (2.41)	7 (4.22)	0.722	1 (1.67)	6 (3.17)	1.000
Chronic liver disease	9 (9.38)	20 (9.85)	1.000	8 (9.64)	14 (8.48)	0.814	5 (8.33)	17 (9.04)	1.000
Coronary artery disease	36 (37.50)	71 (34.80)	0.699	29 (34.94)	59 (35.54)	1.000	19 (31.67)	69 (36.51)	0.538
Diabetes mellitus	18 (18.75)	30 (14.71)	0.400	13 (15.66)	23 (13.86)	0.706	9 (15.00)	27 (14.29)	0.837
Others^a^	21 (21.88)	52 (25.49)	0.565	17 (20.48)	44 (26.51)	0.350	10 (16.67)	51 (26.98)	0.122
Co-contaminant nephrotoxic drugs, n (%)									
Colistin	6 (6.25)	34 (16.67)	0.017	6 (7.23)	25 (15.06)	0.103	3 (5.00)	28 (14.81)	0.045
Aminoglycoside	2 (2.08)	2 (0.98)	0.595	1 (1.20)	2 (1.20)	1.000	0 (0.00)	3 (1.59)	1.000
Amphotericin B	5 (5.21)	5 (2.45)	0.299	3 (3.61)	5 (3.01)	1.000	2 (3.33)	6 (3.17)	1.000
Furosemide	27 (28.13)	70 (34.31)	0.354	23 (27.71)	55 (33.13)	0.469	18 (30.00)	60 (31.75)	0.874
Piperacillin/Tazobactam	11 (11.46)	24 (11.76)	1.000	7 (8.43)	15 (9.04)	1.000	6 (10.00)	16 (8.47)	0.794
Baseline Scr, mean ± SD, mg/dL	1.29 ± 1.30	1.93 ± 1.75	0.002	1.30 ± 1.31	1.93 ± 1.79	0.004	1.32 ± 1.26	1.85 ± 1.76	0.033
Duration of vancomycin therapy, mean ± SD, d	9.64 ± 5.16	10.47 ± 6.01	0.245	9.52 ± 5.22	9.76 ± 4.96	0.723	8.52 ± 4.40	9.97 ± 4.78	0.037
Duration of hospitalization, mean ± SD, d	36.35 ± 25.02	34.32 ± 18.90	0.437	35.05 ± 24.58	34.45 ± 18.94	0.833	34.3 ± 18.02	34.76 ± 21.82	0.882
APACHE II score, mean ± SD	11.74 ± 5.50	13.90 ± 5.38	0.002	12.04 ± 5.56	13.65 ± 5.25	0.026	12.52 ± 5.49	13.30 ± 5.36	0.327
Albumin	3.01 ± 0.63	2.89 ± 0.80	0.184	3.05 ± 0.61	2.91 ± 0.82	0.151	3.02 ± 0.63	2.93 ± 0.78	0.430
MIC of vancomycin for *Enterococcus* spp.									
≤0.5 μg/ml	13 (13.54)	37 (18.14)	0.778	11 (13.25)	31 (18.67)	0.648	0 (0.00)	42 (22.22)	0.001
1.0 μg/ml	78 (81.25)	157 (76.96)		67 (80.72)	127 (76.51)		48 (80.00)	146 (77.25)	
2.0 μg/ml	4 (4.17)	7 (3.43)		4 (4.82)	5 (3.01)		8 (13.33)	1 (0.53)	
4.0 μg/ml	1 (1.04)	3 (1.47)		1 (1.20)	3 (1.81)		4 (6.67)	0 (0.00)	

a Other, gout, hyperthyroid, anemia; APACHE, acute physiology and chronic health evaluation; Scr, serum creatinine; SD, standard deviation.

The 30-days mortality rate was 15.00%. A crude comparison of the two groups reveals that, at 30 days, 16.67 and 14.22% of patients in the vancomycin trough level <15 mg/L and vancomycin trough level ≥15 mg/L groups had died, respectively (*p* = 0.359). The overall rate of clinical failure was 17.33%, seen in 21.88 and 15.20% of the patients in the vancomycin trough level <15 mg/L and vancomycin trough level ≥15 mg/L groups, respectively (*p* = 0.074). The rate of microbiological failure observed was 7.67%, seen in 12.50 and 5.39% of the patients in the vancomycin trough level <15 mg/L and vancomycin trough level ≥15 mg/L groups, respectively (*p* = 0.028) (Table 2).

**TABLE 2:** Cox regression analysis of primary and secondary outcomes for enterococcal infection patients with vancomycin trough concentration ≥15 mg/L.

Variable	Before propensity score matched					
	C_trough_ < 15 mg/L, n (%)	C_trough_ ≥ 15 mg/L, n (%)	Crude HR (95% CI)	*p*-Value	Adjusted HR^a^ (95% CI)	*p*-value
Efficacy primary outcome						
30-days mortality	16 (16.67)	29 (14.22)<	0.75 (0.41–1.39)	0.359	0.50 (0.26–0.95)	0.035
Secondary outcomes						
Clinical failure	21 (21.88)	31 (15.20)	0.60 (0.34–1.05)	0.074	0.53 (0.30–0.96)	0.035
Microbiological failure	12 (12.50)	11 (5.39)	0.40 (0.18–0.91)	0.028	0.28 (0.12–0.69)	0.006
Safety						
Nephrotoxicity	8 (8.33)	53 (25.98)	2.77 (1.31–5.84)	0.001	2.90 (1.36–6.19)	0.006
AKI based on KDIGO Classification						
Stage 1	4 (5.79)	18 (8.82)				
Stage 2	4 (5.79)	19 (9.31)				
Stage 3	0 (0)	15 (7.35)				

HR, hazard ratio; CI, confidence interval.

a Adjusted for all variables with *p* values of < 0.20 and for age and gender.

### Unmatched Cohort Analyses

The multivariate regression analysis showed that vancomycin trough concentrations of ≥15 mg/L were associated with a significant decrease in 30-days mortality (aHR: 0.50, 95% CI: 0.26–0.95; *p* < 0.035), clinical failure (aHR: 0.53, 95% CI: 0.30–0.96; *p* < 0.035), and microbiological failure (aHR: 0.28, 95% CI: 0.12–0.69; *p* = 0.006). However, vancomycin trough concentrations of ≥15 mg/L substantially increase the risk of nephrotoxicity (aHR: 2.90, 95% CI: 1.36–6.19; *p* = 0.006) compared to vancomycin trough concentrations <15 mg/L (Table 2).

### Propensity-Matched Cohort Analyses

Results from the PS matching analysis were comparable to those seen in the unmatched analysis using Cox’s proportional hazards model. Vancomycin trough concentrations of ≥15 mg/L significantly decreased 30-days mortality (aHR: 0.41, 95% CI: 0.21–0.82; *p* < 0.011), clinical failure (aHR: 0.49, 95% CI: 0.26–0.94; *p* < 0.032), and microbiological failure (aHR: 0.32, 95% CI: 0.12–0.87; *p* = 0.025). However, vancomycin trough concentrations of ≥15 mg/L substantially increase the risk of nephrotoxicity (aHR: 3.17, 95% CI: 1.39–7.23; *p* = 0.006) compared to vancomycin trough concentrations <15 mg/L (Table 3). Moreover, a vancomycin AUC/MIC of 389 mg*hr/L was associated with a significant reduction in 30-days mortality (aHR: 0.36, 95% CI: 0.17–0.75; *p* = 0.007), clinical failure (aHR: 0.45, 95% CI: 0.22–0.91; *p* = 0.026), and microbiological failure (aHR: 0.35, 95% CI: 0.13–0.93; *p* = 0.035). However, a vancomycin AUC/MIC of 389 mg*hr/L significantly increased the risk of nephrotoxicity (aHR: 2.89, 95%CI: 1.04–8.01; *p* = 0.041) compared to a vancomycin AUC/MIC of 389 mg*hr/L (Table 3).

**TABLE 3:** Analysis of primary and secondary outcomes for enterococcal infection patients with vancomycin trough concentration ≥15 mg/L and AUC/MIC ≥ 389 mg^a^hr/L.

Outcomes	After propensity score matched							
	Vancomycin trough concentrations				Vancomycin AUC/MIC_vitek_			
	C_trough_ <15 mg/L, *n* = 83	C_trough_ ≥15 mg/L, *n* = 166	Adjusted HR^a^ (95%CI)	*p*-value	AUC/MIC <389 mg^a^hr/L *n* = 60	AUC/MIC≥ 389 mg^a^hr/L *n* = 189	Adjusted HR^a^ (95%CI)	*p*-value
Efficacy Primary outcome								
30-days mortality, n (%)	16 (19.28)	21 (12.65)	0.41 (0.21–0.82)	0.011	12 (20.00)	25 (13.23)	0.36 (0.17–0.75)	0.007
Secondary outcomes								
Clinical failure, n (%)	19 (22.89)	23 (13.86)	0.49 (0.26–0.94)	0.032	13 (21.67)	29 (15.34)	0.45 (0.22–0.91)	0.026
Microbiological failure, n (%)	10 (12.05)	8 (4.82)	0.32 (0.12–0.87)	0.025	8 (13.33)	10 (5.29)	0.35 (0.13–0.93)	0.035
Safety								
Nephrotoxicity, n (%)	7 (8.43)	43 (25.90)	3.17 (1.39–7.23)	0.006	6 (10.00)	44 (23.28)	2.89 (1.04–8.01)	0.041

a Adjusted for all variables with *p* values of < 0.20 and for age and gender.

**FIGURE 3:**
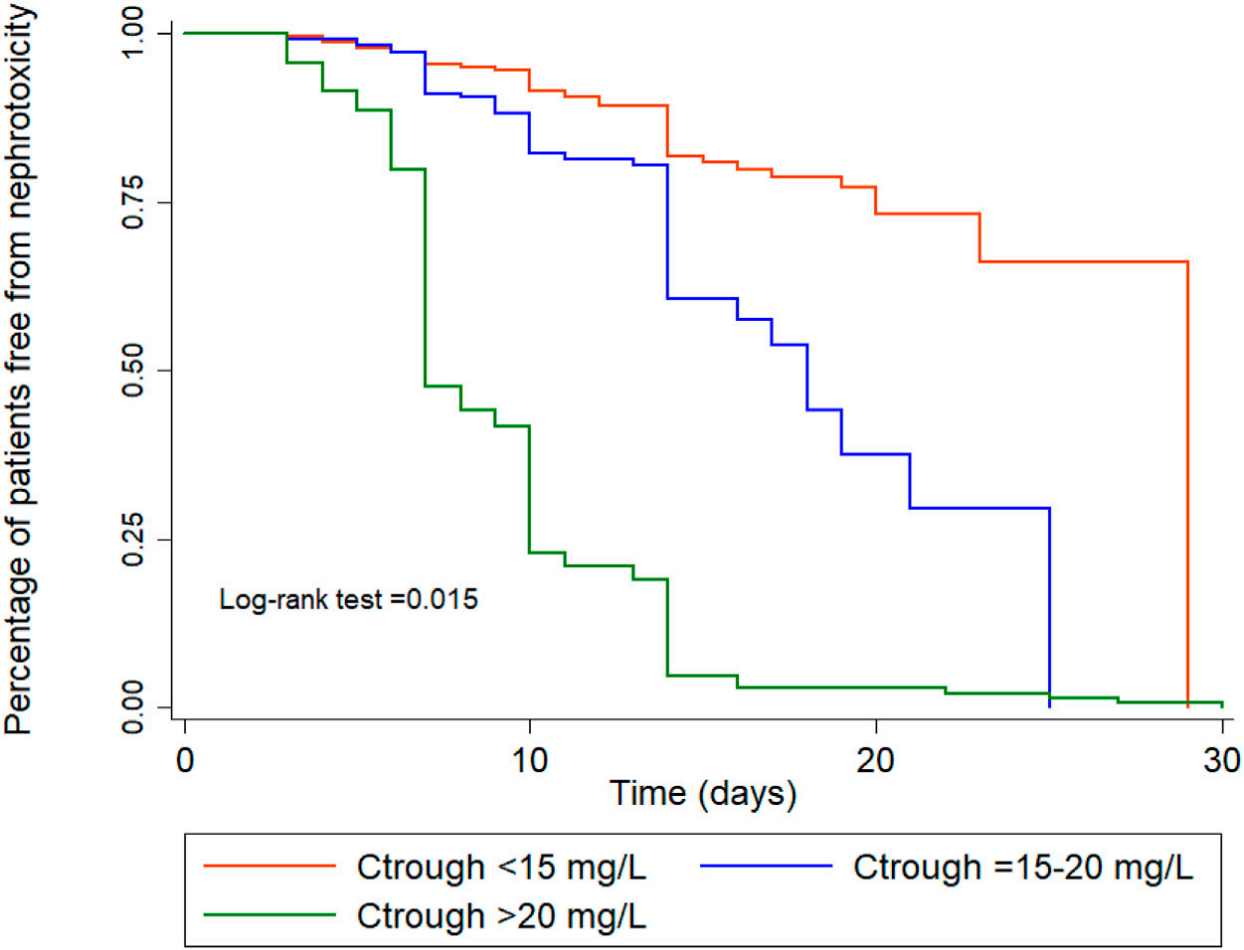
Kaplan-Meier survival analysis for nephrotoxicity for the three trough concentration groups, adjusted for age, gender, APACHE II score, albumin, species, colistin, chronic kidney disease, baseline Scr.

**FIGURE 4:**
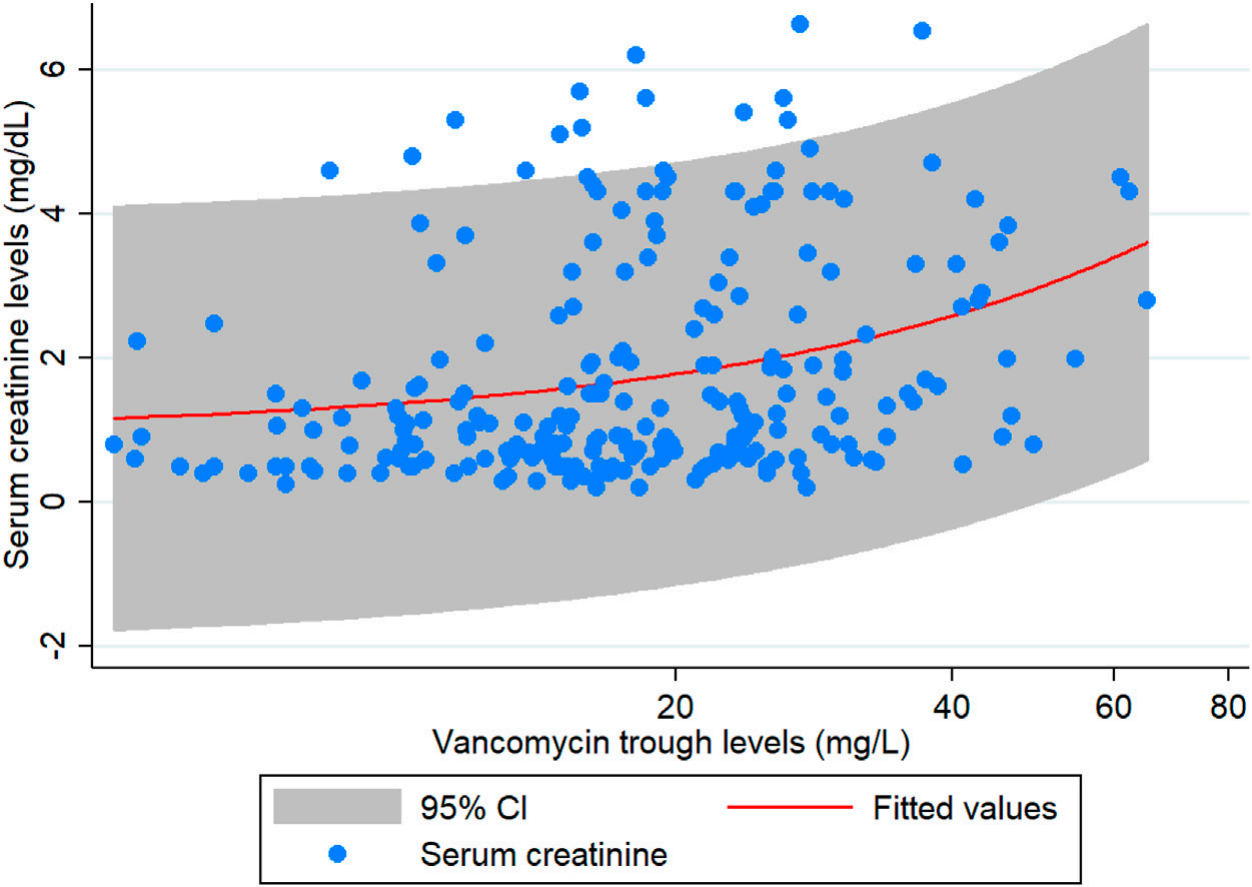
The association between vancomycin trough concentrations and increased serum creatinine.

The results of the subgroup analysis of nephrotoxicity after propensity score matching showed that, when the trough level increased from <15 mg/L to 15–20 mg/L, the rate of nephrotoxicity was significantly increased 2.84-fold (CI: 1.15–6.98, *p* = 0.023). The rate of nephrotoxicity was significantly increased 3.55-fold (CI: 1.57–8.07, *p* = 0.002) higher in patients with trough level of >20 mg/L when compared with the trough levels <15 mg/L. The majority of patients (>90%) with a trough level of >20 mg/L had nephrotoxicity in ≥15 days (Figure 3). These data are shown in Table 4 and Figure 3. Moreover, the association between vancomycin trough concentrations and an increasing serum creatinine are shown in Figure 4.

**TABLE 4:** Subgroup of nephrotoxicity in patients infected with *Enterococcus* spp. classified by vancomycin trough concentrations.

Vancomycin trough concentrations	Before propensity score matched				Propensity score matched	
	Crude HR (95%CI)	*p*-value	Adjusted HR^a^ (95%CI)	*p*-value	Adjusted HR^a^ (95%CI)	*p*-value
C_trough_ < 15 mg/L (*n* = 95)	1 (reference)		1 (reference)		1 (reference)	
C_trough_ 15–20 mg/L (*n* = 78)	2.42 (1.05–5.63)	0.039	2.88 (1.21–6.88)	0.017	2.84 (1.15–6.98)	0.023
C_trough_ > 20 mg/L (*n* = 127)	3.01 (1.40–6.50)	0.005	3.28 (1.49–7.18)	0.003	3.55 (1.57–8.07)	0.002

a Adjusted for all variables with *p* values of < 0.2 and for age and gender.

### Risk Factors Associated With 30 days Mortality Among all Patients With Enterococcal Infections

In the multivariable analysis, treatment of *Enterococcus* infections in patients with the trough levels ≥15 mg/L (aHR, 0.43; 95% CI, 0.22–0.86; *p* = 0.017) was significantly lower of 30 days mortality compared to the trough levels <15 mg/L group (Table 5). Moreover, we observed that the group with 30 days mortality was significantly higher proportion of solid tumor (40.54%; *p =* 0.028), and a higher mean APACHE II score (mean APACHE II score, 15.86; *p* = 0.004).

**TABLE 5:** Multivariable Cox regression model for significant predictors of risk factors for 30-days mortality among all patients with enterococcal infections.

Variable^a^	Survivors (*n* = 212)	Non-survivors (*n* = 37)	aHR (95% CI)^b^	*p* Value
Ctrough ≥ 15 mg/L	145 (68.40)	21 (56.76)	0.43 (0.22–0.86)	0.017
APACHE II score	12.03 ± 4.79	15.86 ± 5.36	1.10 (1.03–1.18)	0.004
Male	91 (42.92)	14 (37.84)	0.57 (0.27–1.19)	0.135
Age	86.67 ± 19.35	64.21 ± 14.53	1.01 (0.99–1.03)	0.315
Albumin	2.96 ± 0.62	2.92 ± 0.77	1.07 (0.72–1.59)	0.748
Enterococcal species	171 (80.66)	31 (83.78)	0.96 (0.39–2.39)	0.932
Colistin	23 (10.85)	8 (21.62)	2.25 (0.99–5.14)	0.054
Chronic kidney disease	69 (32.55)	12 (32.43)	1.06 (0.48–2.36)	0.885
Solid tumor	41 (19.34)	15 (40.54)	2.19 (1.09–4.40)	0.028

a Other factors that were evaluated but did not remain in the stepwise backward regression model included urinary tract infection, bacteremia, wound infection, intra-abdominal infection, respiratory disease, cerebrovascular disease, hematologic malignancy, chronic kidney disease, chronic liver disease, coronary artery disease, diabetes mellitus, aminoglycoside, amphotericin B, furosemide.

## Discussion

In patients with infections due to *Enterococcus,* a vancomycin trough level of ≥15 mg/L was associated with a favorable 30-days mortality rate for enterococcal infection (aHR: 0.41, 95% CI: 0.21–0.82), lower clinical failure rate (aHR: 0.49, 95% CI: 0.26–0.94), and lower microbiological failure rate (aHR: 0.32, 95% CI: 0.12–0.87), when compared with vancomycin trough concentrations <15 mg/L. By contrast, higher rates of nephrotoxicity were associated with a vancomycin trough level of ≥15 mg/L. The usefulness of vancomycin trough level ≥15 mg/L has been highlighted by a recent clinical study in patients with infections due to *Enterococcus*. However, patients with a vancomycin trough level of >20 mg/L had the highest rates of nephrotoxicity. Based on our result, we suggest to achieve vancomycin trough concentration of ≥15 mg/L while try not to exceed 20 mg/L to avoid excessive nephrotoxicity.

Vancomycin therapeutic drug monitoring (TDM) is progressively more frequently utilized clinically (Deryke and Alexander, 2009; Matsumoto et al., 2013). It is imperative that vancomycin treatment is monitored appropriately to reduce the emergence of bacterial resistance (Fernández de Gatta et al., 1996; Zelenitsky et al., 2011). However, the emergence of vancomycin-resistant *enterococci* was not detected in our study.

Our study provides additional evidence on the relationship between vancomycin trough levels and clinical outcomes and nephrotoxicity, addressing some of the concerns of the authors of the consensus review. There is still no specific recommendation for target trough concentration monitoring in the patients of enterococcal infection. However, the recommended minimum trough concentration by 2009 vancomycin guideline (Deryke and Alexander, 2009) is 15 mg/L for treating MRSA infection, 15 mg/L was therefore chosen as the cutoff point. Moreover, Kullar et al. (2011b) conducted a retrospective cohort analysis comprising 320 patients being treated for MRSA bacteremia shortly after the 2009 vancomycin recommendations for sustained trough concentrations of 15–20 mg/L for treatment of MRSA infection (Deryky and Alexander, 2009). According to the study, patients with an initial trough level of <15 mg/L had a 2-fold increased chance of treatment failure, and the risk of nephrotoxicity did not become substantial until trough levels were larger than 20 mg/L. The findings of this study have been used to back up the suggestions in the guideline. So, the patients in our study were divided into two groups by trough concentrations of <15 and ≥15 mg/L.

In general, the virulence of *enterococci* is lower than that of organisms such as *S aureus*. Moreover, MRSA has been reported to be associated with high mortality rates up to 60% (Cosgrove et al., 2003). However, the patients with *E. faecium* bacteremia have a mortality rate of 25.0–34.6% (Gudiol et al., 2013; Billington et al., 2014; Pinholt et al., 2014; Zhang et al., 2017), which is higher than the 30-days all-cause mortality rate (15%) in our study, which included patients with enterococcal infection treated only with vancomycin.

A retrospective study by Nakakura et al. (2019) included 45 patients who were diagnosed with *E. faecium* bacteremia. This study did not compare mortality between high and low trough concentrations. However, they reported the trough concentration among patients who died within 30 days was significantly higher compared to that among patients who survived. This study was retrospective, with a relatively low sample size, which can lead to unfounded associations. Furthermore, the range of drug concentrations seen in the two groups was quite wide and overlapped between the groups, resulting in a lack of definite conclusions as to the exact drug levels affecting the outcome (Nakakura et al., 2019). In addition, the mortality rates between the groups in this study were analysed by crude comparison without adjusting for confounding factors (Nakakura et al., 2019).

Another study featured a retrospective single-center cohort of 57 patients hospitalised with *Enterococcus* bacteremia between January 1, 2009 and May 31, 2015 (Jumah et al., 2018). The primary outcome measure was 30-days mortality. The median concentration of vancomycin troughs was not significantly different between survivors and non-survivors (11.4 mg/L versus 8.6 mg/L*; p* = 0.56). However, this study compared relatively low trough concentration values to values no more than 15 mg/L, so the difference in mortality between groups could not be seen.

However, both previous studies have been performed in relatively small groups of patients. As a result, the power is relatively low and is not sufficient to assess any difference between a vancomycin trough concentration and the mortality rates (Jumah et al., 2018; Nakakura et al., 2019).

Our study found that reduced mortality was associated with a vancomycin AUC/MIC value of ≥389 mg*hr/L. These findings are consistent with the results of a retrospective single-center cohort of 57 patients hospitalised with *Enterococcus* bacteremia between January 1, 2009 and May 31, 2015 [9]. The average vancomycin AUC_24_ was computed using a Bayesian method and found that reduced mortality was correlated with a vancomycin AUC/MIC_Etest_ value of ≥389 mg*hr/L, achieved within 72 h of vancomycin therapy [9]. However, a retrospective study by Nakakura et al. [10] found that, in patients with *E. faecium* bacteremia, there was no substantial difference between mortality in the proportion of patients with vancomycin AUC_24_/MIC ≥389 and <389 mg*hr/L. Moreover, the AUC_24_/MIC ratio was not associated with mortality in patients with *E. faecium* bacteremia. However, the severity of the disease was correlated with mortality in these patients [10].

The recently published of our previous study (Katip and Oberdorfer, 2021) to assess an association between vancomycin AUC/MIC ratio and clinical outcomes and nephrotoxicity in patients with enterococcal infections. The average vancomycin AUC_0–24_ was calculated using a Bayesian approach. A vancomycin AUC/MIC of ≥400 mg*hr/L was related with significant differences in clinical response when compared to a vancomycin AUC/MIC of <400 mg*hr/L (aHR: 0.50, 95% CI: 0.26–0.97; *p* = 0.042). Similarly, compared to a vancomycin AUC/MIC of <400 mg*hr/L, a vancomycin AUC/MIC of ≥400 mg*hr/L was related with significant differences in the microbiological response (aHR: 0.37, 95%CI: 0.14–0.94; *p* = 0.036). Nephrotoxicity was higher in patients with a vancomycin AUC/MIC of ≥400 mg*hr/L (aHR: 3.96, 95 percent CI: 1.09–14.47; *p* = 0.037) than in those with a vancomycin AUC/MIC of <400 mg*hr/L.

The population of our previous study (Katip and Oberdorfer, 2021) was included a population that only complete a 2-point concentration and performed between January 2010 and October 2020. The most common of patients in our previous study was measured 2-point concentration of vancomycin in 2020 after the guideline recommends using AUC-guided dosage and monitoring instead of using a trough-only approach (Katip and Oberdorfer, 2021). However, the present study was performed between January 2010 and December 2019. The population of the present study was used single trough concentration (trough-only measurements). So, the present study was performed in a different population of our previous study (Katip and Oberdorfer, 2021) and not a duplicate patient population. A flowchart of the study population was shown in Figure 1.

Moreover, the differences between the current study and previous our study are compared the two methods (vancomycin trough concentration 15 mg/L and AUC/MIC 389 mg*hr/L) and found that both were associated with a lower 30-days mortality rate, clinical outcomes, microbiological findings, and nephrotoxicity (Tables 3, 6). Thus, before additional randomized studies are carried out, a vancomycin trough value of ≥15 mg/L may be an ideal alternative to the use of AUC values.

**TABLE 6:** Analysis of efficacy and safety of patients with vancomycin trough concentration ≥15 mg/L compared to AUC/MIC ≥ 389 mg*hr/L.

Outcomes	C_trough_ ≥ 15 mg/L, *n* = 166	AUC/MIC ≥ 389 mg*hr/L *n* = 189	*p*-value
Efficacy primary outcome			
30-days mortality, n (%)	21 (12.65)	25 (13.23)	1.000
Secondary outcomes			
Clinical failure, n (%)	23 (13.86)	29 (15.34)	0.764
Microbiological failure, n (%)	8 (4.82)	10 (5.29)	1.000
Safety			
Nephrotoxicity, n (%)	43 (25.90)	44 (23.28)	0.621

In general, nephrotoxicity is common in patients treated with vancomycin, with the most important adverse effects described in the current literature (Deryke and Alexander, 2009; Lodise et al., 2009; Bosso et al., 2011; Rybak et al., 2020). Lodise et al. (2009) observed that higher vancomycin trough levels are associated with higher rates of nephrotoxicity, seeing a 5% AKI risk with initial troughs of <10 mg/L, compared to 21% with troughs of 10–15 mg/L, 20% with 15–20 mg/L, and 33% with troughs of >20 mg/L (Lodise et al., 2009). So, the prevalence of vancomycin-related acute kidney injury (AKI) varies from 5 to 33% (Lodise et al., 2009; Prybylski, 2015), based on a patient survey, trough concentration, and the degree of renal failure (Lodise et al., 2009; Bosso et al., 2011; Prybylski, 2015). In our analysis, increased vancomycin trough levels have been found to be either the cause of nephrotoxicity, or to dramatically increase nephrotoxicity. This is consistent with the results of a multicenter trial conducted at seven hospitals in South Carolina, where they prospectively examined the relative frequency of nephrotoxicity and its relationship with the trough concentration of patients with reported MRSA infections (Bosso et al., 2011). Nephrotoxicity was reported in 42 patients (29.6%) with vancomycin trough concentrations >15 mg/ml and 13 (8.9%) with trough concentrations <15 mg/ml. In this population, multivariate analysis showed that vancomycin trough concentrations >15 mg/ml and ethnicity (black) are risk factors for nephrotoxicity. Vancomycin trough concentrations >15 mg/ml are associated with a three-fold higher risk of nephrotoxicity (Bosso et al., 2011). Moreover, a meta-analysis of 15 studies found that a vancomycin trough >15 mg/L was correlated with 2.67 times of nephrotoxicity rate (95% CI: 1.95–3.65), when compared with <15 mg/L. However, the investigators noticed that, in the majority of cases, nephrotoxicity was reversible and therefore only 3% of nephrotoxic events needed short-term dialysis (van Hal et al., 2013).

Maintaining an appropriate trough level is important not only for achieving the optimum treatment outcome, but also for reducing the incidence of nephrotoxicity. This concern was supported by Fernández de Gatta et al. (1996), who found that achieving optimum trough levels by using TDM of vancomycin led to a reduced incidence of nephrotoxicity (Fernández de Gatta et al., 1996). In our study, the results of the subgroup analysis of nephrotoxicity after propensity score matching showed that, higher trough levels of ≥15 mg/L were associated with higher survival rates than trough levels of <15 mg/L (aHR: 0.41, 95% CI: 0.21–0.82; *p* < 0.011). However, subgroup analysis found that very high trough levels (>20 mg/L) were also associated with a high rate of nephrotoxicity (aHR: 3.55, 95% CI: 1.57–8.07; *p* = 0.002). Furthermore, three patients with AKI are in need of renal replacement therapy.

Some study indicate that in-hospital mortality and infection-attributed hospital stay in *enterococci* blood systemic infection might rather be influenced by *Enterococcus* species and underlying diseases than by vancomycin resistance (Kramer, et al., 2018). Our study observed that the group with 30 days mortality was significantly higher proportion of solid tumor (aHR, 0.43; 95% CI, 0.22–0.86; *p =* 0.028), and a higher mean APACHE II score (aHR, 1.10; 95% CI, 1.03–1.18; *p* = 0.004). Moreover, the patients with trough levels ≥15 mg/L had a lower 30-days mortality rate (aHR, 0.43; 95% CI, 0.22–0.86; *p* = 0.017) than those with trough levels <15 mg/L. However, *Enterococcus* species did not associate with 30 days mortality (aHR, 0.96; 95% CI, 0.39–2.39; *p* = 0.932).

The examination of APACHE II scores is the third component. Additional changes are made for patients with chronic and severe organ failure, including the lungs, heart, liver, kidneys, and immune system. Immunosuppression, chemotherapy, radiation, long-term or high-dose steroids, or severe leukemia, lymphoma, or AIDS are all examples of “immunocompromised” patients.

This large retrospective study offered evidence of the association of clinical outcome and nephrotoxicity with vancomycin trough level in the treatment of enterococcal infection. Although AUC determination has been recommended as a better pharmacokinetic value in order to evaluate clinical results and safety in infections treated with vancomycin (Neely et al., 2018). Moreover, target vancomycin AUC/MIC of ≥400 mg h/L could be optimal for the use for monitoring treatment of enterococcal infections (Katip and Oberdofer, 2021). However, our results indicated that aiming for trough values of ≥15 mg/L may be an ideal alternative to the use of AUC values, before more randomised studies are carried out.

Our study is limited by its retrospective nature, with significant differences in baseline characteristics observed between the treatment groups, making it difficult to compare between the groups and contributing to uncertainty. However, a propensity score-matching approach was used to adjust for established baseline characteristics. The imbalance in bacterial species, baseline Scr, and APACHE II score remained after PS matching but we performed a Cox regression analysis in an effort to resolve the most important confounding factors, ensuring that statistically significant factors of clinical plausibility were retained in our final multivariable model. Secondly, as the study was conducted at a single centre, the results are generalisable only to university hospitals with the same patient populations and vancomycin sensitivity as in our study. Thirdly, the use of all-cause mortality as a primary endpoint for a cohort that has multiple other comorbid conditions which could also be responsible for the patient’s death. However, most retrospective research discovered this confounder. Although we used statistical approaches to control for potential confounders, this study design may still have residual undiscovered confounding factors. Fourthly, this study includes enrolling some *E. facalis* group (most guideline suggest ampicillin) and most infection was catheter-associated urinary tract infection (CA-UTI). Thus, these results do not apply to the other patient groups.

## Conclusion

This study found a significant correlation between vancomycin concentrations of <15 mg/L at initial steady-state and poor clinical outcomes in patients with *enterococcus* infections, such as mortality, clinical failure and microbiological failure, when compared with concentrations of ≥15 mg/L. However, vancomycin trough levels of >20 mg/L were associated with a very high nephrotoxicity rate. Based on our result, we suggest to achieve vancomycin trough concentration of ≥15 mg/L while try not to exceed 20 mg/L to avoid excessive nephrotoxicity. Further studies about optimal vancomycin trough concentration for enterococcal infection will be needed to confirm our findings and help developing professional guidelines.

## Data Availability

The original contributions presented in the study are included in the article/Supplementary Material, further inquiries can be directed to the corresponding author.
